# On the Case of W. O. with Remarks on the Secondary Symptoms of Syphilis

**Published:** 1815-04

**Authors:** John Wilson

**Affiliations:** Devonshire-square


*For the Medical and Physical Journal.*

### *On the Case of W. O. with Remarks on the secondary Symptoms of Syphilis;* by John Wilson, Esq.

HAVING carefully read over the article in your Number for December last, signed W. O., I am induced to offer some observations, which, I trust, if not useful in the particular particular case therein stated, may yet be beneficial in others: at the same time, I must confess it to be my most earnest wish, that these observations may be as salutary to the writer of the article in question as he himself can desire.

Without enumerating, or, perhaps, it being necessary to enumerate, all the circumstances which are related, it may be sufficient to observe, that, in all probability, no one symptom which the unhappy patient laboured under would have occurred, had not syphilitic poison been in the first instance infused into the system; and without, therefore, adverting to the contending opinions which are stated to have been given, as to the proximate or immediate cause of those symptoms, it is certainly of much importance to bear in mind, that upon the first attack, although mercury was exhibited in various forms, and that even though 32 grains of corrosive sublimate were taken in three months, no effect was produced on the mouth. We are not here informed what other preparations of this medicine were employed, or in what form, whether by rubbing or otherwise; but this I may venture to state freely, that 32 grains of corrosive sublimate *may* be taken during the space of three months, not only without producing any effect on the salivary glands, but without producing that cure which is so much to be desired.

Farther, it has very often occurred to me, as I have no doubt it has to other practitioners, that when chancres have been healed by the exhibition of as great quantities of mercury as the patient's constitution could at that time bear, and the symptoms apparently removed, yet, after the lapse of several months, fresh symptoms have occurred, such as nodes on the cylindrical bones, ulcerated throat, distressing nocturnal head-aches, &c. and which have all yielded to moderate doses of the same remedy, especially when continued a sufficient length of time, and due regard being paid to the circumstances of the patient.

To proceed, however, to the secondary or latter stage of this gentleman's complaints, when, in consequence of the excessive misery, pain, emaciation, and want of rest, he felt obliged to have recourse to large doses of laudanum, it appears to me, that, whatever temporary relief might be experienced from this medicine, not only could no cure be expected from it when taken alone, but that it even must ultimately break down the constitution (if I may be allowed that phrase), and this in proportion to the increased strength of the dose.

It is not, therefore, to be wondered at, that in the weakened state of the digestive organs, or primæ viæ, which its use must must have occasioned, that two or three blue pills should have produced griping, tenesmus, and vomiting. I am also strongly inclined to believe that the attack of insensibility, and other epileptic symptoms which are described, were in a great measure owing to the same cause; but here we cannot forbear calling to mind the account which the patient gives of his free living, and which, it may be, perhaps, not unfairly presumed, was continued as long as it could be. In this state of things, it was certainly the best advice that could be given the gentleman, to use a restorative or strengthener, whether in the shape of a balsam or otherwise, together with any nourishing diet which may tend to invigorate the system, but which of themselves would, in my mind, by no means produce a permanent cure.

Having stated thus much, it will naturally be supposed that I have formed some opinion of the nature and cause of the disease, and its present distressing circumstances. In order to do this, it appears to me that we must go back to the original circumstance (viz. syphilitic infection), which was the origin of the whole, and which was not counteracted by its known antidote (viz. mercury) being carried so far into the system as to affect the salivary glands, and thereby to produce a cure.

Again, it does not appear, that at any period of the latter and more afflicting stage of the disease, mercury, with the exception of the two or three blue pills, had been at all employed. It will necessarily follow, if this be correct, that the whole of the symptoms are syphilitic, and that they are only to be cured by the usual means employed for the cure of that disease.

Nevertheless, it by no means follows that mercury in any form, or without being combined with, or assisted by, other medicines, is to be used, or is likely to effect a cure. In the course of more than twenty years' practice, I have met with several cases of the secondary symptoms of syphilis, including all those which have been enumerated above, and which have all yielded to the following remedy, viz. one grain of corrosive sublimate, and one of opium, taken at bed-time only.

It may appear extraordinary, that I should recommend the use of opium, after what I have said above respecting laudanum; or even that I should recommend corrosive sublimate in an emaciated state of the constitution. But here I beg leave particularly to observe, that I have seen cases where opium alone has been given with a view to relieve pain, not only without advantage, but even with the aggravation vation of all the symptoms; and I have also seen cases of the same description, where the exhibition of mercury alone has been attended with great inconvenience and hazard. In these cases, I have found the above combination of mercury with opium to be eminently useful. In many it has operated in the speediest as well as most beneficial manner, giving the patient, according to his own account, more rest and freedom from pain, on the first night of taking, than had been obtained for weeks. I have afterwards been able to increase the dose of corrosive sublimate to two or even three grains, without the necessity of increasing the quantity of opium, and also, which is of very great consequence, without disturbing the ordinary functions of the system.

The cases to which I now allude were not only ultimately cured, but several years have elapsed since some of them occurred to me, and I have the pleasure of knowing that they continue well. It is not for me to say that the same plan would inevitably cure the unfortunate writer of the article in question; but I would, with great deference, submit to him, or to his medical attendant, a trial of the above remedy.

*Devonshire-square;* JOHN WILSON.

*Feb.* 22, 1815.

